# Analysis of Herpes Simplex Virion Tegument ICP4 Derived from Infected Cells and ICP4-Expressing Cells

**DOI:** 10.1371/journal.pone.0070889

**Published:** 2013-08-06

**Authors:** Suzanne M. Pritchard, Cristina W. Cunha, Anthony V. Nicola

**Affiliations:** 1 Department of Veterinary Microbiology and Pathology, Washington State University, Pullman, Washington, United States of America; 2 Animal Disease Research Unit, USDA-Agricultural Research Service, Pullman, Washington, United States of America; 3 Paul G. Allen School for Global Animal Health, Washington State University, Pullman, Washington, United States of America; McMaster University, Canada

## Abstract

ICP4 is the major transcriptional regulatory protein of herpes simplex virus (HSV). It is expressed in infected cells with immediate early kinetics and is essential for viral growth. ICP4 is also a structural component of the virion tegument layer. Herpesviral tegument proteins exert regulatory functions important for takeover of the host cell. Tegument ICP4 has not been well characterized. We examined the ICP4 present in HSV-1 virions that were either derived from wild type infected cells or from ICP4-expressing (E5) cells infected with ICP4 deletion virus *d*120. Limited proteolysis demonstrated that virion-associated ICP4 from particles derived from E5 cells was indeed an internal component of the virion. A similar subset of virion structural proteins was detected in viral particles regardless of the cellular origin of ICP4. Genotypically ICP4-negative virions complemented with tegument ICP4 entered cells via a proteasome-dependent, pH-dependent pathway similar to wild type virions. In infected cells, ICP4 was distributed predominantly in intranuclear replication compartments regardless of whether it was expressed from a transgene or from the HSV genome.

## Introduction

The herpes simplex virus (HSV) DNA genome is housed in an icosahedral capsid surrounded by an asymmetrical tegument layer and a surface envelope [1]. The tegument contains >19 HSV-encoded proteins and trace amounts of host cell proteins [2]. Herpesvirus tegument components mediate critical processes during the viral life cycle including viral gene expression, capsid transport to and from the nucleus, and acquisition of the virion envelope. During lytic replication, HSV expresses its genes in an orderly cascade. Immediate early (IE) proteins are critical for the expression of the subsequent early (E) and late (L) genes [1].

The viral IE regulatory proteins ICP0 and ICP4 are abundantly expressed in the infected cell [1]. They are also present in the virion tegument layer at 100–200 copies each [3], [4], [5], [6], [7], [8], [9], [10], [11]. Capsid-associated ICP0 has been proposed to regulate transport of the subviral particle to the nucleus during viral entry [10]. Little is known about ICP4 that is brought into the cell with the entering virion. ICP4 is a 175 kiloDalton DNA-binding phosphoprotein that is required for activation of E and L genes [12], [13]. In infected cells at late times post-infection, ICP4 localizes to defined intranuclear compartments where it interacts with components of the cellular RNA polymerase II transcriptional machinery to either activate or repress transcription [14], [15], [16], [17], [18]. ICP4 is absolutely essential for HSV infection, and consequently, viruses that are deleted for ICP4, such as *d*120 [19], must be propagated on ICP4-expressing cells, such as the complementing Vero cell line E5 [20]. Much of our understanding of ICP4 function in HSV infection is based on studies of ICP4-null viruses derived from infected ICP4-expressing cells. In an effort to better understand ICP4 associated with viral particles, we compared tegument ICP4 that originates from the ICP4 complementing cell line E5 with tegument ICP4 derived from infection of Vero cells.

## Materials and Methods

### Cells and Viruses

Vero and U2OS cells (American Type Culture Collection, Rockville, MD) were propagated in Dulbecco modified Eagle medium (Invitrogen, Grand Island, NY) supplemented with 10% fetal bovine serum (Gemini Bio-Products, West Sacramento, CA). CHO-nectin-1 (M3A) cells [21] (kindly provided by Roselyn Eisenberg and Gary Cohen, University of Pennsylvania) are a hamster cell line derived from the parental CHO-K1 hamster cell line. CHO-nectin-1 cells are stably transformed with the human nectin-1 gene and the *Escherichia coli lacZ* gene under the control of the HSV ICP4 promoter [21]. The cells were propagated in Ham F-12 nutrient mixture (Invitrogen) supplemented with 10% fetal bovine serum, 150 µg of puromycin (Sigma, St. Louis, MO)/ml, and 250 µg of G418 sulfate (Fisher Scientific, Fair Lawn, NJ)/ml. Cells were subcultured in nonselective medium prior to use in experiments.

Wild type HSV-1 strain KOS was provided by Priscilla Schaffer (Harvard University). HSV-1 KOS mutant *d*120, containing a 4.1-kb deletion in both copies of the ICP4 gene [19], and the Vero-derived, complementing cell line E5 [20] were kindly provided by Neal DeLuca (University of Pittsburgh, PA). Stocks of *d*120 virus were propagated on E5 cells. Wild-type Glasgow strain 17 syn+ (17+) [22] and its ICP0 deletion mutant derivative *dl*1403 [23] were provided by R. Everett, MRC Virology Unit, Glasgow. 17+ and *dl*1403 were propagated and titered on U2OS cells.

### SDS-PAGE and Western Blot Analysis of Virions

Extracellular HSV-1 virions were prepared by infecting Vero cells grown to 90% confluence with HSV-1 KOS or *d*120 at a multiplicity of infection (MOI) of 0.01. After detection of significant cytopathic effect but prior to complete detachment of the cell monolayer, the virus-containing medium was collected and spun at 1,400×*g* to remove cell debris. Where indicated, supernatant was layered onto a 5% sucrose cushion, and virions were pelleted by centrifugation for 1 h at 27,000×*g*. Samples in Laemmli buffer were separated by SDS polyacrylamide gel (4–20% gradient) electrophoresis. Gels were blotted onto nitrocellulose and probed with 1 µg of mouse monoclonal antibody (MAb)/ml specific for gB, ICP4, VP5 (MAbs H1359; Virusys, Sykesville, MD, HA018, H1A021; Santa Cruz, respectively), ICP0 (MAb 11060; Virusys) or 0.01 µg/ml MAb 1–21 to VP16 (Santa Cruz). Rabbit monoclonal antibody to Rab7 (MAb 9367; Cell Signaling Technology, Danvers, MA) was used at 1∶2000. Anti-HSV rabbit polyclonal antibody (HR50; Fitzgerald Industries, Acton, MA) was added at 25 µg/ml. Nitrocellulose membranes were incubated with horseradish peroxidase-conjugated goat secondary antibody (Pierce, Rockford, IL), developed with enhanced chemiluminescence detection reagents (Pierce), and exposed to X-ray film (Kodak).

### Limited Proteolysis of HSV Particles

To investigate the location of viral proteins relative to virions, extracellular HSV-1 KOS or *d*120 virions were treated with various concentrations of Proteinase K (Sigma) for 15 min on ice. To halt proteolysis, warmed Laemmli buffer was added, and reactions were boiled for 10 min. Samples were analyzed by 4–20% SDS-PAGE and Western blotting.

### Immunofluorescence Microscopy

Virus was added to cell monolayers grown on glass coverslips in 24-well culture dishes at an MOI of 5. At 6 hr post-infection, cultures were fixed in ice-cold methanol and blocked with 1% BSA. 1 µg/ml anti-ICP4 MAb H1A021 was added followed by Alexa 488-labeled goat anti-mouse IgG (Invitrogen). Images were captured with an EVOS FL fluorescence microscope (Life Technologies).

### Treatments with Proteasome Inhibitor or Lysosomotropic Agents

MG132 (75 µM; Sigma) and monensin (75 µM; Sigma) stock solutions were prepared in dimethyl sulfoxide and ethanol, respectively, and stored at **−**20°C. 1.5 M stock solution of ammonium chloride (Sigma) was prepared in water immediately prior to use. Confluent CHO-nectin-1 cell monolayers were grown in 96-well dishes. Growth medium was removed from cells and replaced with medium containing agents or medium containing control concentrations of dimethyl sulfoxide or ethanol. Cultures were incubated for 15 to 30 min at 37°C. Virus was added, and cells were incubated in the constant presence of agent for 6 to 7 h. Entry was measured by beta-galactosidase assay.

### Beta-galactosidase Reporter Assay for HSV Entry

Following infection, 0.5% Nonidet P-40 (Sigma) cell lysates were prepared. Chlorophenol red-beta-D-galactopyranoside (Roche Diagnostic, Indianapolis, IN) was added, and the beta-galactosidase activity was read at 595 nm with an ELx808 microtiter plate reader (BioTek Instruments, Winooski, VT). Beta-galactosidase activity indicated successful entry. Mean results and standard deviations were calculated for four replicate samples. Each experiment was performed at least three times with similar results.

## Results and Discussion

### ICP4 is Incorporated into the Tegument of Virions Lacking the ICP4 Gene

Functional studies of virion proteins are facilitated by the ability to generate viral particles that lack the gene of interest and the protein for which it encodes. For example, functional analysis of tegument ICP0 was made possible by obtaining mutant viral particles that lack virion ICP0 [7]. Infection of cells with an ICP0 deletion mutant virus yields genotypically null virions that also lack tegument ICP0. This is possible due to the dispensable nature of the ICP0 gene at high MOI [24].

**Figure 1:**
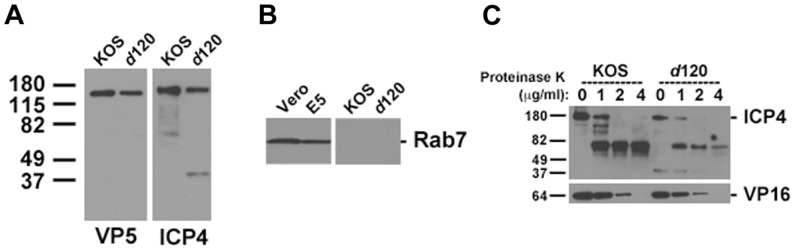
Detection and localization of ICP4 in HSV-1 *d*120 virions propagated on ICP4 complementing cell line E5. Approximately similar amounts of cell-free supernatant preparations, as estimated by densitometry of VP5, of HSV-1 KOS or *d*120 or cell lysates of uninfected Vero or E5 cells were separated by 4–20% SDS-PAGE. Western blots were probed with MAbs to HSV VP5 or HSV ICP4 (A), or cellular Rab7 (B). Results shown are representative of three (A) or two (B) independent experiments. (C) Extracellular HSV-1 KOS or *d*120 virions were treated with the indicated concentrations of Proteinase K for 15 min on ice. Samples were immediately boiled in Laemmli buffer and separated by 4–20% SDS-PAGE. Western blots were probed with MAbs to ICP4 or VP16. Results shown are representative of at least five independent experiments. Molecular weight standards in kiloDaltons are indicated to the left of each panel.

ICP4 is essential for replication, and genotypically null virions must be propagated on a cell that provides ICP4. E5 cells contain the ICP4 gene under the control of the ICP4 promoter, so ICP4 expression is induced upon infection [20]. To determine whether HSV-1 *d*120 propagated on ICP4-expressing cells is truly complemented with tegument ICP4, the mutant virions were analyzed. Approximately similar amounts of *d*120 particles as determined by Western blotting with antibody against VP5 (Fig. 1A) were examined. Relative to VP5, levels of ICP4 similar to those of wild type KOS virions were found associated with *d*120 virions and a ∼ 40 kiloDalton ICP4 fragment was detected as reported previously [25]. To begin to address whether ICP4 is present in *d*120 virion preparations as a cellular contaminant due to ICP4 overexpression by the E5 cells, we probed for the host cell protein Rab7. KOS and *d*120 virions did not contain detectable levels of Rab7 as determined by Western blot (Fig. 1B). The same was true for longer exposures to film (not shown). As expected, Rab7 was detected in Vero and E5 cells (Fig. 1B).

To probe further whether ICP4 was peripherally associated with *d*120 particles or whether it was present inside of the particles, limited proteolysis of HSV-1 *d*120 was performed. Proteinase K treatment of both *d*120 and wild type KOS virions resulted in a similar pattern of ICP4 proteolysis. Partial proteolysis was observed at 1 µg/ml Proteinase K, and virtually complete digestion of full length (175 kiloDalton) ICP4 was observed at 2 µg/ml (Fig. 1C). A control tegument protein, VP16, from either *d*120 or KOS virions was similarly susceptible to Proteinase K (Fig. 1C), thus the ICP4 associated with *d*120 virions is present in the particle interior. The virion-associated ICP4 in *d*120 particles is indeed tegument ICP4, and not the result of contaminating exogenous ICP4 from the E5 cells.

The results suggest that ICP4 expressed from a transgene is incorporated into assembling progeny virions, possibly the tegument. In this regard, virion ICP4 is distinct from virion ICP0. ICP0-null viruses grown on ICP0-expressing cells do not contain tegument ICP0 (unpublished data). This may be due to cell-expressed ICP0 localizing to the nucleus instead of the cytoplasm, which is the site of ICP0 incorporation into progeny virions. The subcellular site of ICP4 recruitment to the virion tegument and whether ICP4 is appropriately localized in E5 cells for proper tegumentation are open questions. To begin to address these issues, we next analyzed the subcellular distribution of ICP4 in ICP4-expressing E5 cells infected with either wild type or ICP4-deletion viruses.

### Localization of ICP4 in HSV-infected Complementing Cells

**Figure 2:**
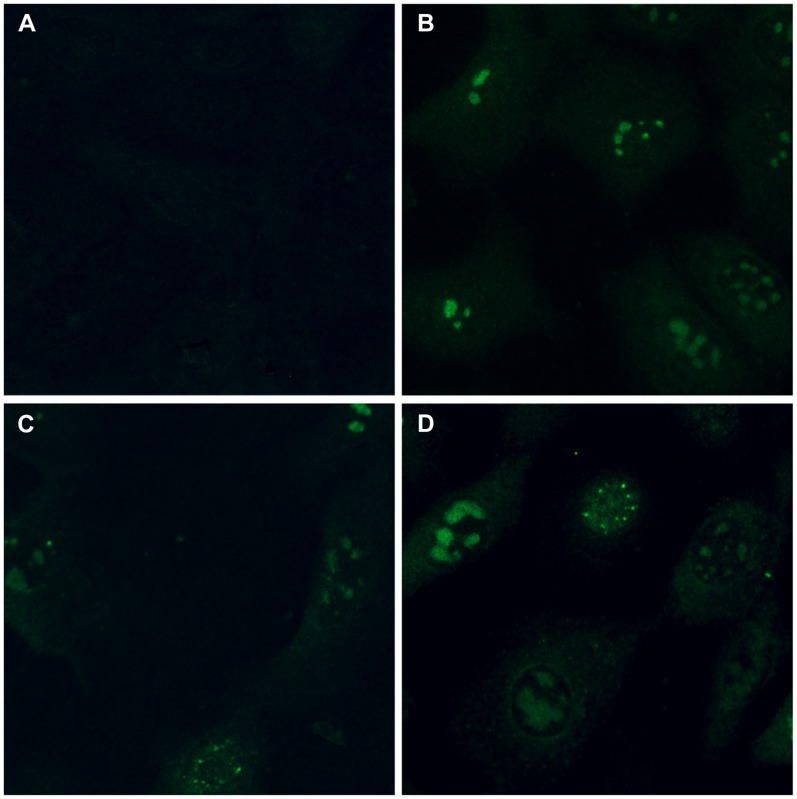
Subcellular distribution of ICP4 in ICP4-expressing cells infected with wild type or *d*120 virus. HSV-1 *d*120 (ICP4-null) (A, C) or KOS (B, D) was added to Vero (A, B) or E5 (ICP4-expressing) (C, D) cell monolayers at an MOI of 5. At 6 hr post-infection, cells were fixed, and ICP4 was detected with MAb H1A021 followed by fluorescent secondary antibody. Results shown are representative of at least three independent experiments with each condition tested in triplicate. Magnification, 40×.

The distribution of ICP4 in a complementing cell line was examined by immunofluorescence microscopy of infected cells. Detectable fluorescence could come from tegument-derived ICP4, newly synthesized ICP4, and/or the cell (in E5 cells). Vero cells were infected with HSV-1 *d*120 at an MOI of 5 for 6 hr. ICP4 fluorescence above background was not observed, indicating that input tegument ICP4 was not detectable under these experimental conditions (Fig. 2A). When E5 cells were infected with KOS or *d*120, ICP4 fluorescence was present predominantly in intranuclear globular replication compartments (Fig. 2C and D). More diffuse, pinpoint nuclear staining of ICP4 was detected in some E5 cells (Fig. 2C and D). This pattern resembles the intranuclear staining for ICP4 in KOS-infected Vero cells at earlier times post-infection prior to the formation of the larger replication compartments [15]. ICP4-specific fluorescence was also detected in the cytoplasm of E5 cells. Dual expression of ICP4 protein from viral and cellular sources (Fig. 2D) results in ICP4 distribution that does not differ greatly from that of wild type-infected Vero cells (Fig. 2B and [14]). The ICP4 fluorescence detected in HSV-1 *d*120-infected E5 cells (Fig. 2C) suggests that ICP4 derived solely from the cellular transgene exhibits wild type subcellular localization. The number of ICP4-positive cells decreased with an MOI of <1 (data not shown). Taken together the results suggest that ICP4 is properly localized in E5-infected cells (Fig. 2C) to allow for successful assembly into the tegument of progeny virions, consistent with wild type levels of incorporation of ICP4 into *d*120 particles (Fig. 1A).

### Protein Composition of HSV ICP4 Mutant Virions Propagated on ICP4-expressing Cells

**Figure 3:**
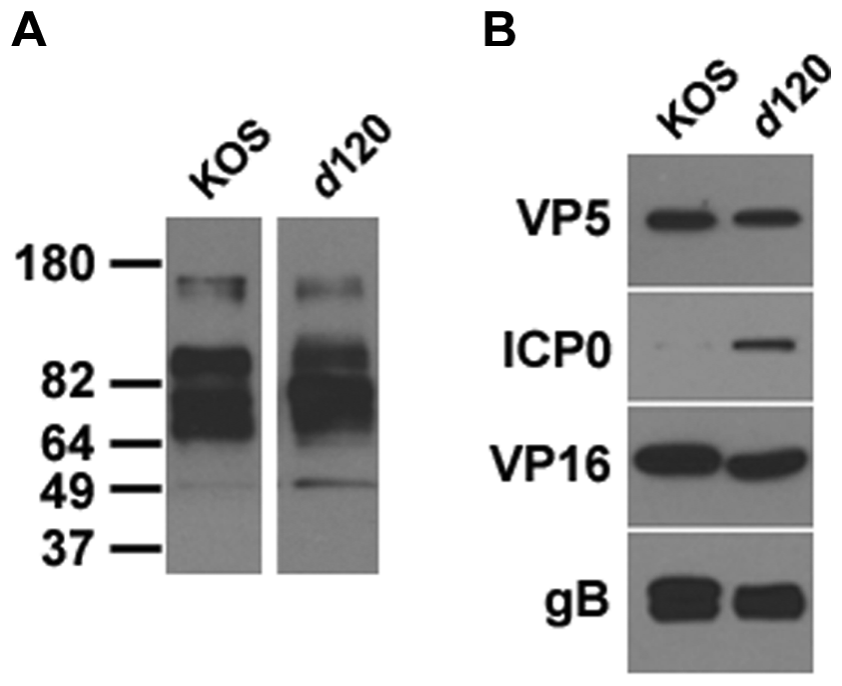
Examination of protein content of HSV-1 *d*120 virions. Equivalent VP5 units of HSV-1 KOS or *d*120 virions were separated by 4–20% SDS-PAGE. Western blots were probed with anti-HSV polyclonal antibody (A) or MAbs to VP5, ICP0, VP16, or gB (B). Results shown are representative of at least three independent experiments. Molecular weight standards in kiloDaltons are indicated to the left.

We investigated whether ICP4 that was expressed from E5 cells and incorporated into tegument (as in Fig. 1) influences the protein composition of mature extracellular virions. Equivalent VP5 units of virions of parental virus HSV-1 KOS or virions of ICP4 null mutant *d*120 were analyzed by SDS-PAGE followed by Western blotting. The ladder of KOS structural proteins that were detectable by the anti-HSV polyclonal antibody was also detected in *d*120 (Fig. 3A). To examine specific representative proteins, equivalent VP5 units of extracellular virions were immunoblotted. Levels of gB and VP16 did not appear to be reduced in the *d*120 particles (Fig. 3B), but interestingly there was a reproducible increase in the amount of ICP0 in *d*120 relative to wild type virions. Thus, tegumentation of *d*120 capsids in ICP4-expressing cells may result in an increased incorporation of ICP0. This is consistent with previous reports of cells infected with ICP4 mutant viruses having ICP0 predominantly in the cytoplasm, the site of its incorporation [14], [26], [27]. Notably, the absence of tegument ICP0 from HSV particles does not affect the levels of ICP4 incorporated [7]. Based solely on the analysis in Figure 3, we cannot rule out that one or more structural proteins may be present in KOS virions but missing from the *d*120 virions or vice-versa. However, for the HSV proteins analyzed, except ICP0, the content of virions released from E5 cells was not grossly altered relative to wild type virions.

#### Proteasome dependence of entry of HSV containing tegument ICP4 from distinct sources

ICP4 expressed in HSV-infected cells as an IE protein has a well-characterized, essential role in viral replication [13]. No function has been ascribed to ICP4 that is present in the virion tegument. Tegument ICP0 appears to play a novel role compared to its cell-expressed IE counterpart in infected cells. During viral entry, HSV interacts with the host cell machinery in a coordinated manner [28], [29]. Virion ICP0 may regulate incoming capsid transport to the nuclear periphery during viral entry [10]. Since ICP0 functionally cooperates with ICP4 in gene expression [30], we interrogated the role of tegument ICP4 in proteasome-dependent entry. Entry of wild type HSV is blocked by MG132, but virions lacking tegument ICP0 are more refractory to inhibition [10], [31].

**Figure 4:**
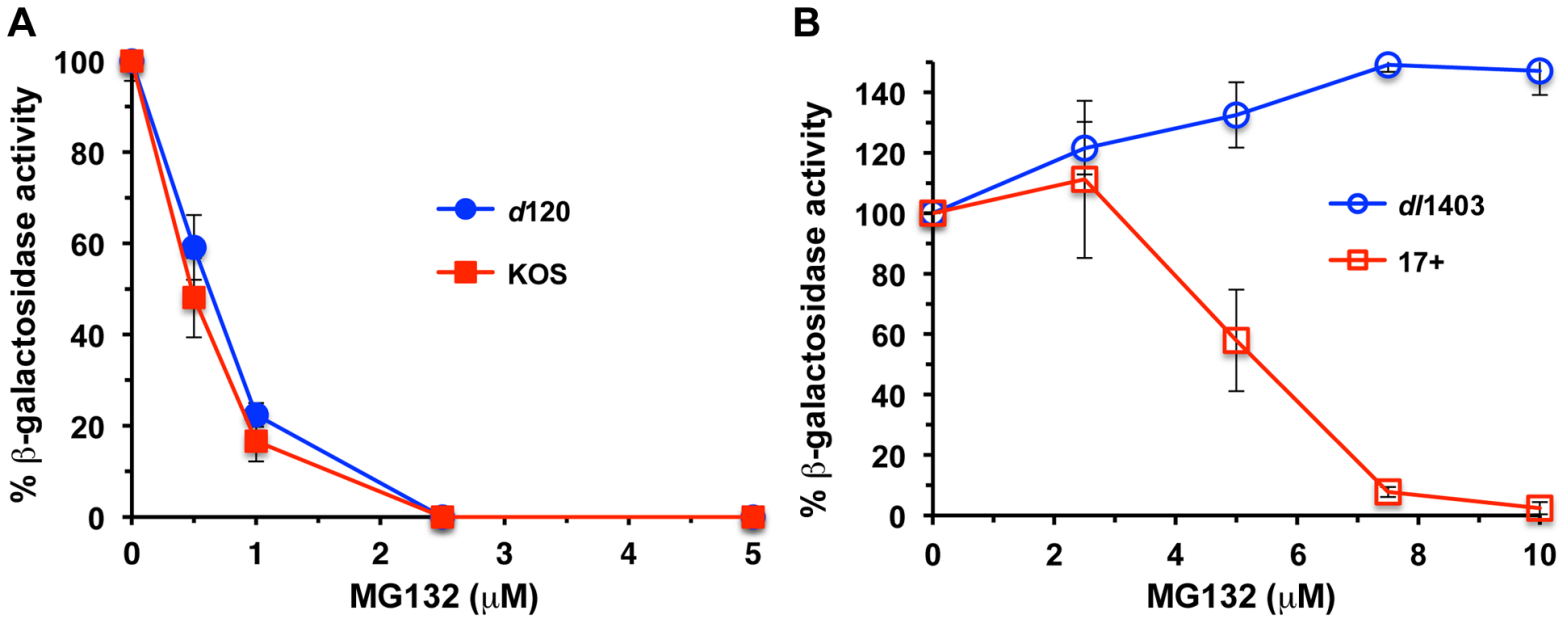
Proteasome-dependence of *d*120 entry into cells. (A) CHO-nectin-1 cells were subcultured in 96-well plates. 20 min prior to infection, cells were treated with the indicated concentrations of MG132. HSV-1 KOS and *d*120 (A) or 17+ and *dl*1403 (B) was added (MOI of 4) for 6 to 7 hr at 37°C in the continued presence of agent. Beta-galactosidase activity indicated successful entry. The percent beta-galactosidase activity relative to that obtained in the absence of MG132 is indicated. The data are means of quadruplicate determinations with standard deviation. Experiments were performed at least three times with similar results. Entry of *d*120 in the presence of MG132 was not statistically different from wild type (p = 0.904, Student's t-test). In contrast, Student's t-test analysis of *dl*1403 entry and its matched wild type yielded a p value of 0.012.

Since KOS virions contain reduced levels of ICP0 relative to *d*120 virions (Fig. 3B), we queried whether inhibition of the proteasome would affect entry of *d*120 virions differently than KOS virions. Entry of *d*120 was inhibited by MG132 in a concentration-dependent manner similar to wild type (Fig. 4A) despite its apparently increased levels of tegument ICP0 (Fig. 3B). The entry of a mutant HSV-1 KOS that is completely devoid of tegument ICP0 is refractory to inhibition by MG132 (Fig. 4B) [10]. Virion ICP0 is capsid-associated and has been proposed to regulate the post-penetration, proteasome-dependent entry of HSV [7], [10]. One explanation for the result in Figure 4A may be that the relatively small amount of ICP0 in wild type KOS virions is sufficient to allow proteasome-dependent entry, and that the increased level of ICP0 present in *d*120 virions does not alter sensitivity to proteasomal inhibition. The phosphorylation state of tegument ICP4 remains to be investigated, as does whether there is a difference in ICP4 phosphorylation based on the source of the protein.

### Entry Pathway taken by HSV Containing Tegument ICP4 from Distinct Sources

**Figure 5:**
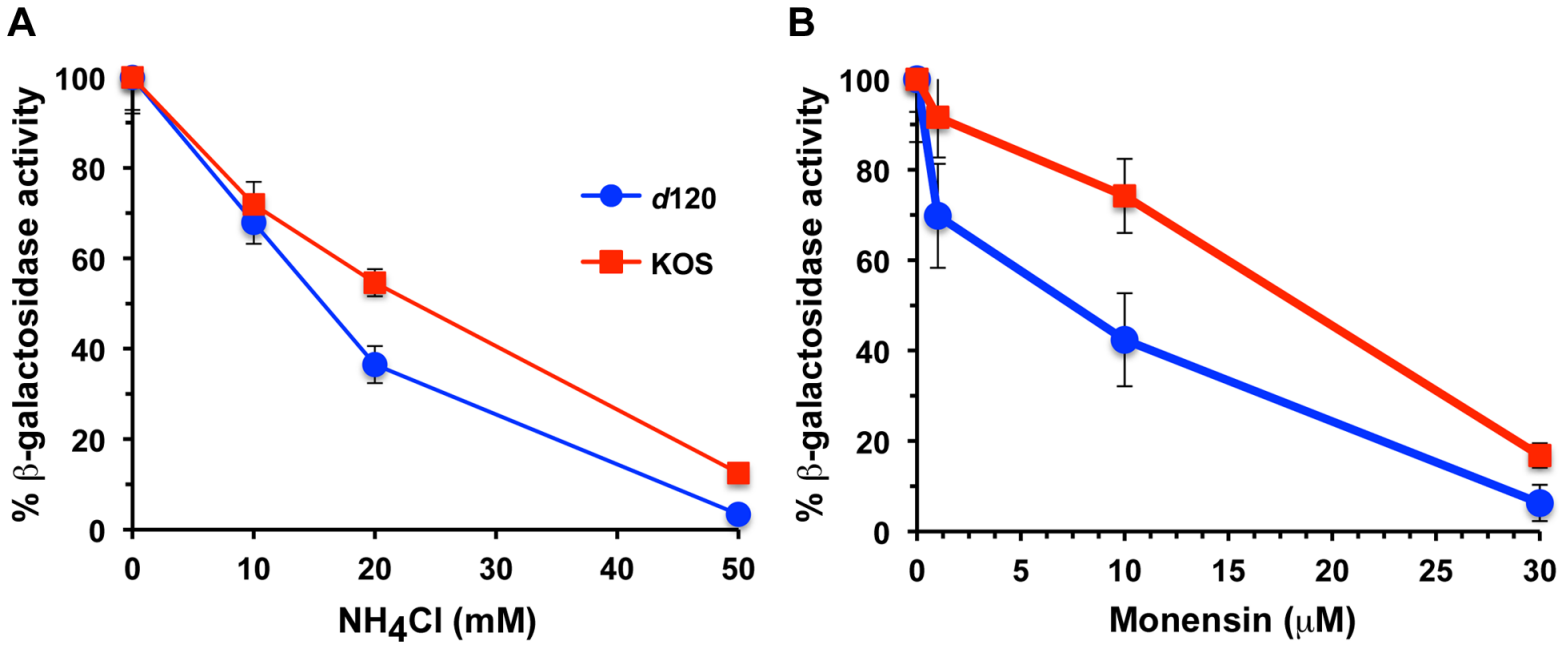
HSV-1 *d*120 enters cells via an acid-dependent endocytosis pathway. CHO-nectin-1 cells were treated with the indicated concentrations of (A) ammonium chloride (NH_4_Cl) or (B) monensin for 20 min at 37°C. Equivalent genome copy numbers of HSV-1 KOS or *d*120 virions were added for 6 to 7 hr at 37°C in the constant presence of agent. The percent beta-galactosidase activity relative to that obtained in the absence of agent is indicated. The data are means of quadruplicate determinations with standard deviation. Experiments were performed at least three times with similar results. Entry of *d*120 in the presence of NH_4_Cl or monensin was not statistically different from KOS wild type (p = 0.787 or p = 0.579, respectively, Student's t-test).

HSV entry requires endosomal low pH in several cell types including CHO-nectin-1 cells [32]. It has been proposed that conformational changes in gB triggered by endosomal pH lead to membrane fusion [33]. To examine whether *d*120 virions utilize a similar entry pathway to wild type virions, the lysosomotropic agents ammonium chloride and monensin were used to determine the pH-dependence of the HSV entry pathway. Entry of *d*120 into CHO-nectin-1 cells was inhibited by both pH-altering agents in a concentration-dependent manner (Fig. 5A–B). HSV-1 *d*120 may have an enhanced sensitivity to inhibition relative to wild type, particularly to monensin (Fig. 5B). Together the data suggest that HSV enters via a pH-dependent endocytic pathway regardless of the origin of virion ICP4.

ICP4 is clearly a component of the HSV tegument layer. It is difficult to address tegument ICP4 function without first generating a viable HSV that lacks tegument ICP4. Study of tegument ICP0 has been greatly facilitated by the isolation of virions that lack ICP0 protein. Here we rigorously demonstrate that propagation of an ICP4-deletion virus on E5 cells does not result in a virus particle free of ICP4 protein. A future hypothesis to be tested is that incoming structural ICP4 regulates transcription in a manner similar to IE-expressed ICP4. Alternately, virion ICP4 may play a purely structural role with no direct function of its own. Our current strategy to generate virions devoid of ICP4 is to identify and ablate the ICP4 packaging signal. This will be difficult if there is overlap in the signal for virion incorporation and regions necessary for ICP4 function. The current study also demonstrates that the cellular origin of ICP4 does not affect its subcellular distribution, nor does it affect the proteasome-dependence or pH-dependence of entry.
